# In Petticoats

**Published:** 1888-03

**Authors:** Leroy Henry


### IN PETTICOATS.

I have had an experience. One afternoon I was dressed in woman's apparel. I was dressed by women because I don't think I could have properly adjusted the various articles. I requested them to dress me as the average woman dresses. When they laced the corset I told them they were making it too tight, but they earnestly claimed they were not making it as tight as average women do. After about a bushel of dry goods were attached to me, I was turned loose to try the realities of woman's dress, and there the trouble began. First, I couldn't breathe well enough, though I kept trying all the time. Second, when I undertook to put on my shoes I couldn't bend enough to reach my feet the first trial. Third, when I walked, I had to push the skirts ahead with my front foot, and that took one-fifth more vital force than would have been required with only pantaloons. Fourth, in carrying two buckets of water up stairs I had to set one bucket down to get a free hand to raise my skirts and tuck them under my elbow, so I could hold them up. Fifth, it took twice as long to climb a fence, and three times the trouble to appear "elegant" and not show my pants. Sixth, while rowing I could not keep my skirts from getting wet and muddy from the bottom of the boat. Seventh, my stomach became heated and soured my dinner.

Why do women wear such clothes as they do? Their dress is a great draw back to their physical and mental development. The tightness about the waist lessens their life force by shutting off part of their breathing capacity, weakens all their powers and renders them an easy prey to the too prevalent female diseases.

Its injuriousness is not equalled by the custom of the heathen Chinese in compressing the feet. Who thinks a wasp waist, a five inch foot, or a flat-head Indian is beautiful? Womankind are surely very tough to do the work and stand the worry they do, and wear tight dresses and long skirts suspended from the hips. Do they want to change and do better? Is popular opinion keeping them from it? Then let them all agree to adopt a change on a certain day and let the change be universal.

Oh, yes, I discovered one important thing. Economical county authorities could use corsets instead of prison cells to punish the bad men. I have never tried the prison cell, but I think the corset would be the greater punishment.

If any man objects to women wearing clothes similar to what he wears let him get his wife or sister to dress him in woman's ordinary dress, and let him wear it for a day and do his usual work, and if he don't change his mind it will be because—because—"A wise man changes his mind, but a fool never." If he wants to get the opinion of eminent health reformers on the subject let him get "Woman's Way out," a little pamphlet advertised in *Lucifer*.

To return to the story. When evening came and I divested myself of my tortuous harness (with the help of an assistant who was better acquainted with the *modus operandi*) I felt free and happy, and I have been glad ever since that I was born a pantaloons-wearing animal instead of a skirt and corset wearing one.—LEROY HENRY, *in Lucifer*.

